# Developmental Differences in Neocortex Neurogenesis and Maturation Between the Altricial Dwarf Rabbit and Precocial Guinea Pig

**DOI:** 10.3389/fnana.2021.678385

**Published:** 2021-05-31

**Authors:** Mirjam Kalusa, Maren D. Heinrich, Christine Sauerland, Markus Morawski, Simone A. Fietz

**Affiliations:** ^1^Faculty of Veterinary Medicine, Institute of Veterinary Anatomy, Histology and Embryology, University of Leipzig, Leipzig, Germany; ^2^Medical Faculty, Paul Flechsig Institute of Brain Research, University of Leipzig, Leipzig, Germany

**Keywords:** precocial, altricial, cortex development, neurogenesis, neuron maturation, dwarf rabbit, guinea pig

## Abstract

Mammals are born on a precocial–altricial continuum. Altricial species produce helpless neonates with closed distant organs incapable of locomotion, whereas precocial species give birth to well-developed young that possess sophisticated sensory and locomotor capabilities. Previous studies suggest that distinct patterns of cortex development differ between precocial and altricial species. This study compares patterns of neocortex neurogenesis and maturation in the precocial guinea pig and altricial dwarf rabbit, both belonging to the taxon of Glires. We show that the principal order of neurodevelopmental events is preserved in the neocortex of both species. Moreover, we show that neurogenesis starts at a later postconceptional day and takes longer in absolute gestational days in the precocial than the altricial neocortex. Intriguingly, our data indicate that the dwarf rabbit neocortex contains a higher abundance of highly proliferative basal progenitors than the guinea pig, which might underlie its higher encephalization quotient, demonstrating that the amount of neuron production is determined by complex regulation of multiple factors. Furthermore, we show that the guinea pig neocortex exhibits a higher maturation status at birth, thus providing evidence for the notions that precocial species might have acquired the morphological machinery required to attain their high functional state at birth and that brain expansion in the precocial newborn is mainly due to prenatally initiating processes of gliogenesis and neuron differentiation instead of increased neurogenesis. Together, this study reveals important insights into the timing and cellular differences that regulate mammalian brain growth and maturation and provides a better understanding of the evolution of mammalian altriciality and presociality.

## Introduction

The neocortex is a highly complex and organized structure of the mammalian brain, which has undergone considerable expansion and specialization during evolution. It consists of six horizontal neuronal layers with two major types of neurons: glutamatergic projection neurons (~70–85%), born in the dorsal telencephalon and GABAergic interneurons (~15–30%), originating from the ventral telencephalon (Hendry et al., 1987; Letinic et al., 2002; Marin and Rubenstein, 2003; Wonders and Anderson, 2006; Han and Sestan, 2013; Hansen et al., 2013). Projection neurons primarily arise during embryonic and fetal development and originate from two major classes of neural progenitor cells (NPCs): apical progenitors (APs) and basal progenitors (BPs) (Fietz and Huttner, 2011; Florio and Huttner, 2014; Molnar et al., 2014; Dehay et al., 2015; Cardenas and Borrell, 2020; Kalebic and Huttner, 2020). APs are the primary NPCs whose cell body resides in the ventricular zone (VZ), the germinal zone that lines the lateral ventricle. They possess apical cell polarity and a radially oriented basal process and characteristically express the marker protein Pax6 (Supplementary Figure 1) (Rakic, 1972; Aaku-Saraste et al., 1997; Chenn et al., 1998; Götz et al., 1998; Miyata et al., 2001; Gal et al., 2006; Kosodo et al., 2008; Marthiens and Ffrench-Constant, 2009). APs divide at the ventricular surface. Before the onset of neurogenesis, APs mostly undergo symmetric proliferative divisions, thereby laterally expanding the VZ (Rakic, 1995). With the onset of neurogenesis, most APs start dividing asymmetrically, thereby generating BPs that accumulate in the subventricular zone (SVZ), the germinal zone basal to the VZ (Haubensak et al., 2004; Miyata et al., 2004; Noctor et al., 2004). BPs lack apical cell polarity and consist of two major subtypes: basal intermediate progenitors and basal radial glia (Supplementary Figure 1) (Fietz et al., 2010; Hansen et al., 2010; Reillo et al., 2011). Basal intermediate progenitors retract their apical and basal processes before M-phase and characteristically express the marker protein Tbr2 (Englund et al., 2005; Attardo et al., 2008). They represent the major BP cell type in rats and mice, in which they mainly undergo symmetric neurogenic (consumptive) division, thus displaying limited proliferative potential (Supplementary Figure 1A) (Attardo et al., 2008). Basal radial glia represent the major BP cell type in mammals that exhibit a high degree of neocortex expansion, e.g., primates including macaque and human. Besides lacking an apical domain, basal radial glia share major features with APs, including the expression of Pax6 and a radially oriented process throughout the cell cycle, and can undergo repeated cell division (Fietz et al., 2010; Hansen et al., 2010; Reillo et al., 2011; Betizeau et al., 2013). In contrast to rats and mice, a major proportion of primate basal intermediate progenitors is characterized by sustained expression of Pax6 and a high proliferate potential (Supplementary Figure 1B). Together, this results in a more expanded SVZ and a higher neuronal output in primates, particularly in human (Fietz et al., 2010; Hansen et al., 2010; Betizeau et al., 2013; Florio and Huttner, 2014; Gertz et al., 2014; Ostrem et al., 2017; Cardenas and Borrell, 2020).

Newborn neurons of the developing dorsal telencephalon migrate radially into the CP in a birth date-dependent *inside*–*out* manner. Later-born neurons form the more superficial layers and migrate past earlier-born neurons that form the deeper layers (Angevine and Sidman, 1961; Rakic, 1974, 1988). As they mature, they gain morphological and electrophysiological characteristics required to attain their functional state enabling an animal's cognitive, sensory, and locomotor abilities. This involves the extension of an axon and dendrites and the establishment of appropriate input and output connectivity and requires the presence of external support and supply structures, i.e., involving glial cells for the provision of nutrients and oxygen, the formation of blood–brain barrier, the maintenance of extracellular (ion) milieu and the myelination of extensions (Moe et al., 2005; Wolpert et al., 2007; Hammond, 2008).

Mammals are born on a precocial-altricial continuum. The offspring of mammalian species at the altricial end of the altricial–precocial continuum are generally hairless, have closed distant organs, are incapable of locomotion, and depend on heat and food from their mother at birth and only become independent late in life. In contrast, the offspring of mammalian species at the precocial end of the altricial–precocial continuum generally possess a well-developed coat and functional sensory organs, are capable of locomotion, require little warmth and food from the mother at birth, and are relatively independent at an early age (Dieterlen, 1963; Martin and Maclarnon, 1985; Derrickson, 1992; Werneburg et al., 2016). Previous data suggest that the pattern of prenatal neurogenesis and brain maturation differs between precocial and altricial species (Maslova and Ozirskaia, 1979; Dudine and Gozzo, 1983; Brunjes, 1984; Gozzo et al., 1985; Pintor et al., 1986; Tessitore and Brunjes, 1988; Tombol, 1988; Brunjes et al., 1989; Lossi et al., 2002; Charvet and Striedter, 2011). Specifically, the higher cognitive, sensory, and locomotor capabilities of precocial— in contrast to altricial—species at birth might indicate higher prenatal neurogenesis and brain maturation (Glatzle et al., 2017). However, a detailed direct comparison of brain development between precocial and altricial species lacks until now. It is, therefore, the aim of this project to compare patterns of brain development, specifically neocortex neurogenesis and maturation, in phylogenetically closely related precocial and altricial species; i.e., the domestic guinea pig (Dunkin Hartley strain) of the order Rodentia and the domestic rabbit (colored dwarf rabbit) of the order Lagomorpha, both belonging to the taxon of Glires (Fox, 1980) and diverging from a common ancestor ~73 million years ago (Supplementary Figure 2) (Upham et al., 2019).

The domestic rabbit (*Oryctolagus cuniculus f. domestica*) is an altricial species belonging to the family Leporidae of the order Lagomorpha (Fox, 1980; Varga, 2014). Colored dwarf rabbits have an adult body weight of 1,000–1,500 g (Thormann, 2012) and a gestation period of 30–32 days, after which five to eight young with a birth weight of 40–100 g are born (Varga, 2014). The domestic guinea pig (*Cavia porcellus f. domestica*) is a precocial species belonging to the family Caviidae of the order Rodentia (Hückinghaus, 1961; Wagner and Manning, 1976; Frye and Hedges, 1995; Harkness et al., 2002; Suckow et al., 2012). Dunkin*-*Hartley guinea pigs have an adult body weight of 995–1,442 g (McDougall et al., 2009). After a gestation period of 63–70 days, mostly three young with a birth weight of 52–131 g (Peaker and Taylor, 1996; Kapoor and Matthews, 2005; Rocca and Wehner, 2009). Both species are herbivores, live in a similar habitat with hierarchical social behavior (King, 1956; Kunkel and Kunkel, 1964; Wagner and Manning, 1976; Sachser, 1998; Asher et al., 2004; Varga, 2014) and possess a lissencephalic brain (Zilles et al., 2013).

Our data demonstrate that the basic order of neuro- and gliogenesis as well as dendrite and axon formation and myelination of extensions are preserved in the neocortex of both species analyzed. Furthermore, we show that neurogenesis starts at a later postconceptional time point and lasts longer in the neocortex of the guinea pig than the dwarf rabbit and that the developing dwarf rabbit neocortex seems to be characterized by a higher abundance of highly proliferative BPs when compared with that of the guinea pig, thus indicating that the amount of neuron production is determined by complex regulation of multiple factors including the duration of the neurogenesis period and the absolute and relative number of distinct NPCs. Furthermore, our findings show that the newborn guinea pig neocortex exhibits a higher maturation status than the dwarf rabbit, suggesting that precocial species have acquired the morphological machinery required to attain their high functional state at birth and that brain expansion observed in precocial newborn might be largely due to prenatally initiating processes of gliogenesis and neuron maturation. Together, our findings provide new insights into the timing and cellular differences that regulate mammalian brain growth and maturation and a greater understanding of the evolutionary mechanisms involved in the process of speciation within the altricial–precocial spectrum.

## Materials and Methods

### Brain Samples

Developing brain tissue from 18 guinea pigs and 19 dwarf rabbits was used in the study. Guinea pigs were obtained from Charles River, housed and mated at the MEZ (Medizinisch-Experimentelles Zentrum, Faculty of Medicine, University of Leipzig). Time-pregnant dwarf rabbits were obtained from the Tierarztpraxis Dr. Falko Pötzsch (Eilenburg/Wurzen, Germany) and private breeding facilities (Blankenfelder Zwerge, Nina Pülmer, Blankenfelde-Mahlow; Christine Sauerland, Leipzig). The age of the guinea pigs ranged from prenatal day 15 to 60 and is stated as days post conception (p.c.) (d15 p.c., *n* = 2; d20 p.c., *n* = 2; d25 p.c., *n* = 2; d31 p.c., *n* = 3; d40 p.c., *n* = 3; d50 p.c., *n* = 3; d60 p.c., *n* = 3). The age of the dwarf rabbits ranged from prenatal (PE) day 15 to postnatal (PO) day 30 (PE15, *n* = 3; PE20, *n* = 3; PE25, *n* = 3; PE30, *n* = 2; PO5, *n* = 2; PO10, *n* = 2; PO20, *n* = 2; PO30, *n* = 2). The time of birth was estimated to occur at day 30 p.c. in the dwarf rabbit (Varga, 2014), and all age information is stated as days p.c. Pregnant dams and pups were killed by intraperitoneal injection with pentobarbital (1 mL/kg). All experiments were performed following German animal welfare legislation and were approved by Landesdirektion Leipzig (T 50/14, 48/16, 11/19).

Animals were carefully dissected. For d15–25 p.c., complete embryos/fetuses were fixed immediately in 4% paraformaldehyde (PFA) for at least 4 days. For d30–60 p.c., fetuses and pups were carefully dissected, brains were fixed in 4% PFA for 7 days at 4°C. After fixation, all brains were weighted, washed in phosphate-buffered saline (PBS), and stored in pH 7.4 buffered 0.02% PBS azide at 4°C.

### Immunocytochemistry

Brain samples were processed and subjected to an immunohistochemistry protocol described previously (Sauerland et al., 2018). In brief, fixed hemispheres were cryoprotected in 30% sucrose in PBS at room temperature until they sank to the bottom, embedded in Tissue-Tek (Sakura Finetek, AJ Alphen aan den Rijn, Netherlands), and stored at −20°C. Complete telencephalon was cut coronally at 30 μm using a cryostat. Sections at a medium position concerning the rostrocaudal axis were heated for 1–1.5 h at 90–98°C in 0.01 M citrate buffer (pH 6), permeabilized with 0.3% Triton X-100 in PBS and quenched with 0.1 M glycine. Primary antibodies were incubated overnight at 4°C, and secondary antibodies were incubated for 1 h at room temperature. The following primary antibodies were used: Tbr1 (1:100, rabbit, Millipore, Darmstadt, Germany, AB10554), Pax6 (1:100, rabbit, Biolegend, London, United Kingdom, 901301), Tbr2 (1:100, sheep, R&D Systems, Abingdon, United Kingdom, AF6166), Hu C/D (1:100, rabbit, Abcam, Amsterdam, Netherlands, ab184267), neurofilament H (1:250, rabbit, Abcam, Amsterdam, Netherlands, ab8135), MAP2 (1:100, chicken, Abcam, Amsterdam, Netherlands, ab5392), MBP (1:100, rat, Abcam, Amsterdam, Netherlands, ab7349), GFAP (1:250, rabbit, antibodies.com, Cambridge, United Kingdom, A85419), and synaptophysin (1:500, mouse, Invitrogen, Darmstadt, Germany, MA1-213). Donkey secondary antibodies coupled to Alexa rb555 (A31572) (1:500, life technologies, Darmstadt, Germany) and Alexa chicken 488 (A11039), m488 (A21202), rat 488 (A21208), sh647 (A21448) (1:500, Invitrogen, Darmstadt, Germany) were used. All sections were counterstained with DAPI (1:500, Sigma, Taufkirchen, Germany), mounted in Mowiol (Merck Biosciences, Darmstadt, Germany), coverslipped, and kept at 4°C.

### Image Acquisition and Analysis

Fluorescence images were acquired using a Leica SP8 confocal laser-scanning microscope, using a 40 × /1.1 objective. Images were acquired as single optical sections. All images were processed using Fiji 2 and Photoshop CS6 software (Adobe). The VZ, SVZ, intermediate zone (IZ)/subplate (SP), and cortical plate (CP) were identified based on their cytoarchitecture as described previously (Sauerland et al., 2018). In brief, the VZ was identified as a densely packed cell layer that lines the lateral ventricle and whose nuclei exhibit radial morphology. The SVZ was identified as a cell layer adjacent to the VZ that exhibits a looser and sparser cell arrangement than the VZ. The intermediate zone (IZ)/subplate (SP) was identified as a cell layer between the SVZ and the cortical plate (CP) that exhibit a very low cell density. The CP was identified as a densely packed cell layer adjacent to the IZ/SP.

Quantification of cells was performed using Fiji software using a Multiclass Cell Counter plug-in (Schindelin et al., 2012). Positive nuclei for the parameters indicated were counted in rectangular sectors of the cortex spanning its entire thickness (Hu C/D) or the thickness of the VZ or SVZ (Pax6, Tbr2). They are expressed as the number of cells per 100 μm ventricular surface. In addition, the fluorescence signal of single channels was counted using grayscale color and an adjusted threshold. The same rater performed all quantifications on images from the dorsal-lateral telencephalon. The radial thickness of the cortical plate and the length of the ventricular surface were determined by tracing it in Fiji software. Data were further analyzed using Prism software (GraphPad Software, San Diego, USA).

NPC cell counts (i.e., Pax6+/Tbr2+, Pax6+/Tbr2–, and Pax6-/Tbr2+ NPCs in the VZ and SVZ) and CP thickness were compared between the dwarf rabbit and guinea pig at corresponding cortical neurogenesis stages using two-tailed unpaired Student's *t*-test, *p*-values below 0.05 were considered significant. Corresponding developmental stages were determined according to Workman et al. (2013) (www.translatingtime.org) and were as follows: d15 p.c. (dwarf rabbit) and d25 p.c. (guinea pig), d20 (dwarf rabbit) and d 31 p.c. (guinea pig), d30 p.c. (dwarf rabbit) and d40 p.c. (guinea pig), d40 p.c. (dwarf rabbit) and d50 p.c. (guinea pig), d50 p.c. (dwarf rabbit), and d60 p.c. (guinea pig).

To extrapolate the timing of neurogenesis, the development of Pax6+ and Tbr2+ NPCs in the VZ and SVZ was plotted in Prism (GraphPad Software) assuming Gaussian distribution. As a measure of goodness of fit, *R*^2^ was calculated. Encephalization quotient (EQ) was calculated according to Boddy et al. (2012): EQ = brain mass/(0.56 × body mass ^∧^0.746). Index of Neural Development (IND) was calculated as adapted from (Portmann, 1990) and (Grand, 1992): IND = developmental brain mass/adult brain mass. Data on the developmental brain weight of both species were obtained in this study. For dwarf rabbits, adult brain and body weight data were obtained from the literature (Latimer and Sawin, 1955). For guinea pigs, data of adult brain weight were obtained in this study, data of adult body weight were obtained from the literature (Dobbing and Sands, 1970).

The phylogenetic tree was constructed using the MammalTree service from vertlife.org. The following species were chosen from the provided list: *Rattus norvegicus, Mus musculus, Hydrochoerus hydrochaeris, Cavia porcellus, Oryctolagus cuniculus, Lepus europeus, Homo sapiens, Macaca mulatta, Callithrix jacchus, Sus scrofa, Equus caballus, Felis silvestris*. The tool first trims the phylogenetic data to a subset, then samples the tree from a chosen pseudo-posterior distribution and provides the tree for downloading. The pruned tree was plotted with FigTree1.4.4 (http://tree.bio.ed.ac.uk/) and adapted in Illustrator CS6 software (Adobe, San Jose, California).

## Results

### Developmental Differences in Neurogenesis Between the Dwarf Rabbit and Guinea Pig Neocortex

**Figure 1:**
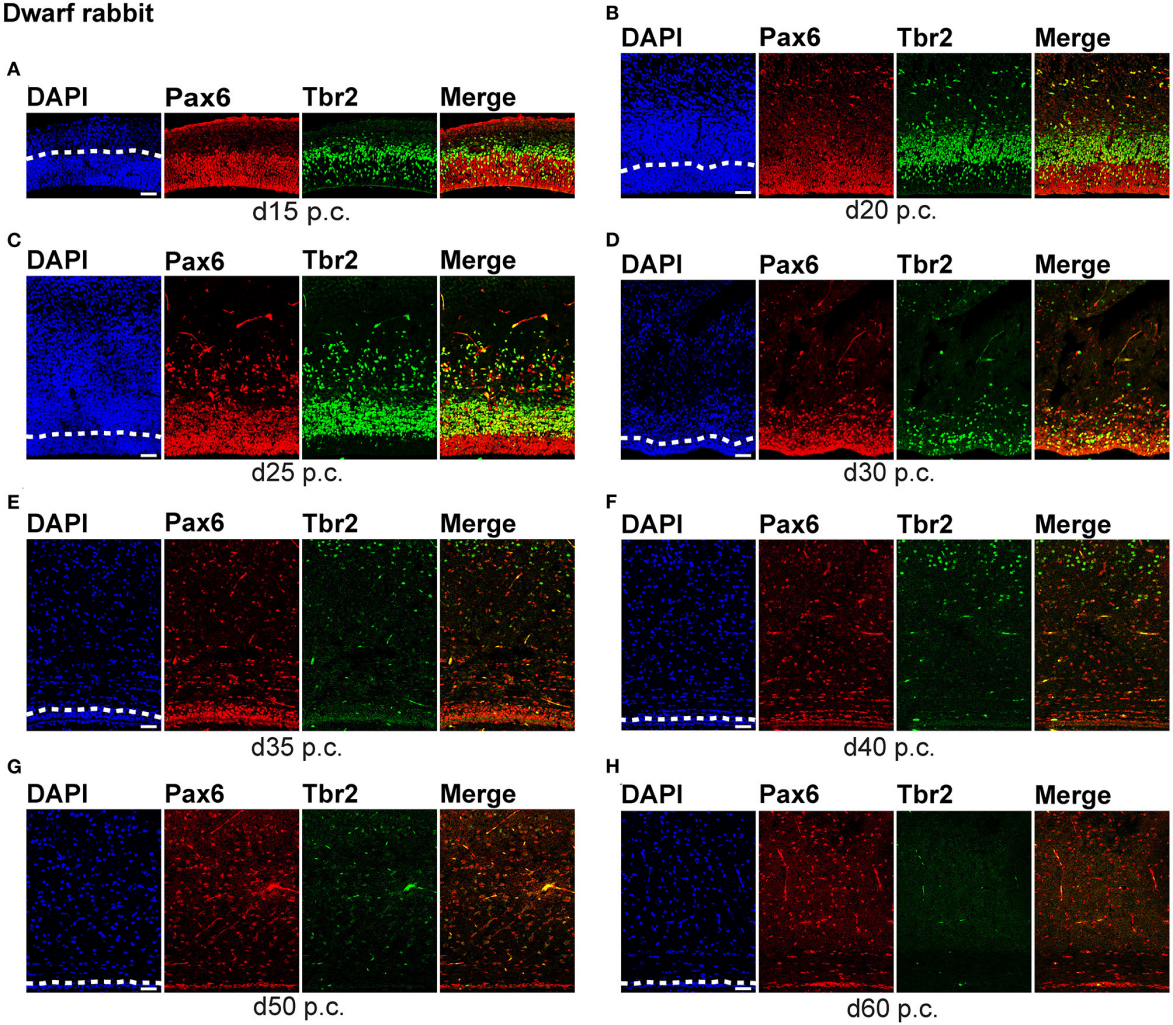
Pax6 and Tbr2 expression in the germinal zones of the developing dwarf rabbit neocortex. **(A–H)** Double immunofluorescence for Pax6 (red) and Tbr2 (green) and DAPI staining (blue) on 30-μm cryosections of day 15 to 60 post-conception (p.c.) dwarf rabbit neocortex. The merge images show combined immunofluorescence for Pax6 and Tbr2. **(A)** The complete cortical wall is shown. **(B–H)** The top margin of the image corresponds to the transition zone SVZ/intermediate zone **(B,C)** or the intermediate zone **(D–H)**. The dashed line indicates the border between VZ and SVZ. Scale bars, 50 μm. VZ, ventricular zone; SVZ, subventricular zone.

**Figure 2:**
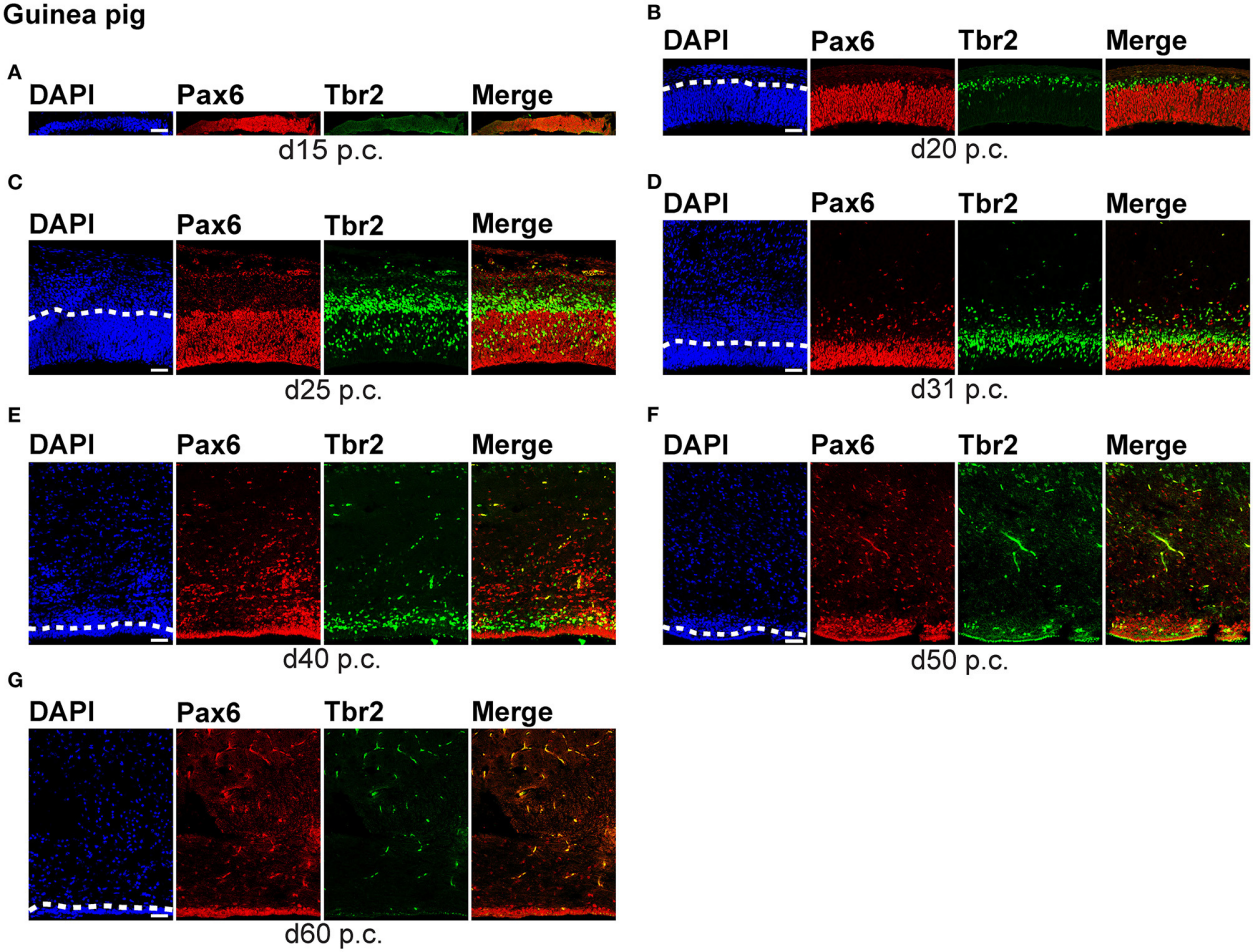
Pax6 and Tbr2 expression in the germinal zones of the developing guinea pig neocortex. **(A–G)** Double immunofluorescence for Pax6 (red) and Tbr2 (green) and DAPI staining (blue) on 30-μm cryosections of day 15 to 60 post-conception (p.c.) guinea pig neocortex. The merge images show combined immunofluorescence for Pax6 and Tbr2. **(A–C)** The complete cortical wall is shown. **(D–G)** The top margin of the image corresponds to the transition zone SVZ/intermediate zone **(D)** or the intermediate zone **(E–G)**. Scale bars, 50 μm. The dashed line indicates the border between VZ and SVZ. VZ, ventricular zone; SVZ, subventricular zone.

To compare specific aspects of neocortex neurogenesis between altricial and precocial mammalian species, we first focused our analysis on the characterization of the distinct NPCs, specifically on their occurrence and abundance, and analyzed cortical sections of the dwarf rabbit and guinea pig from different developmental stages by immunohistochemistry for the expression of Pax6 and Tbr2, both known to be characteristically expressed by distinct NPC subtypes (Figures 1, 2, Supplementary Figure 1) (Götz et al., 1998; Englund et al., 2005; Fietz et al., 2010; Hansen et al., 2010; Reillo et al., 2011).

**Figure 3:**
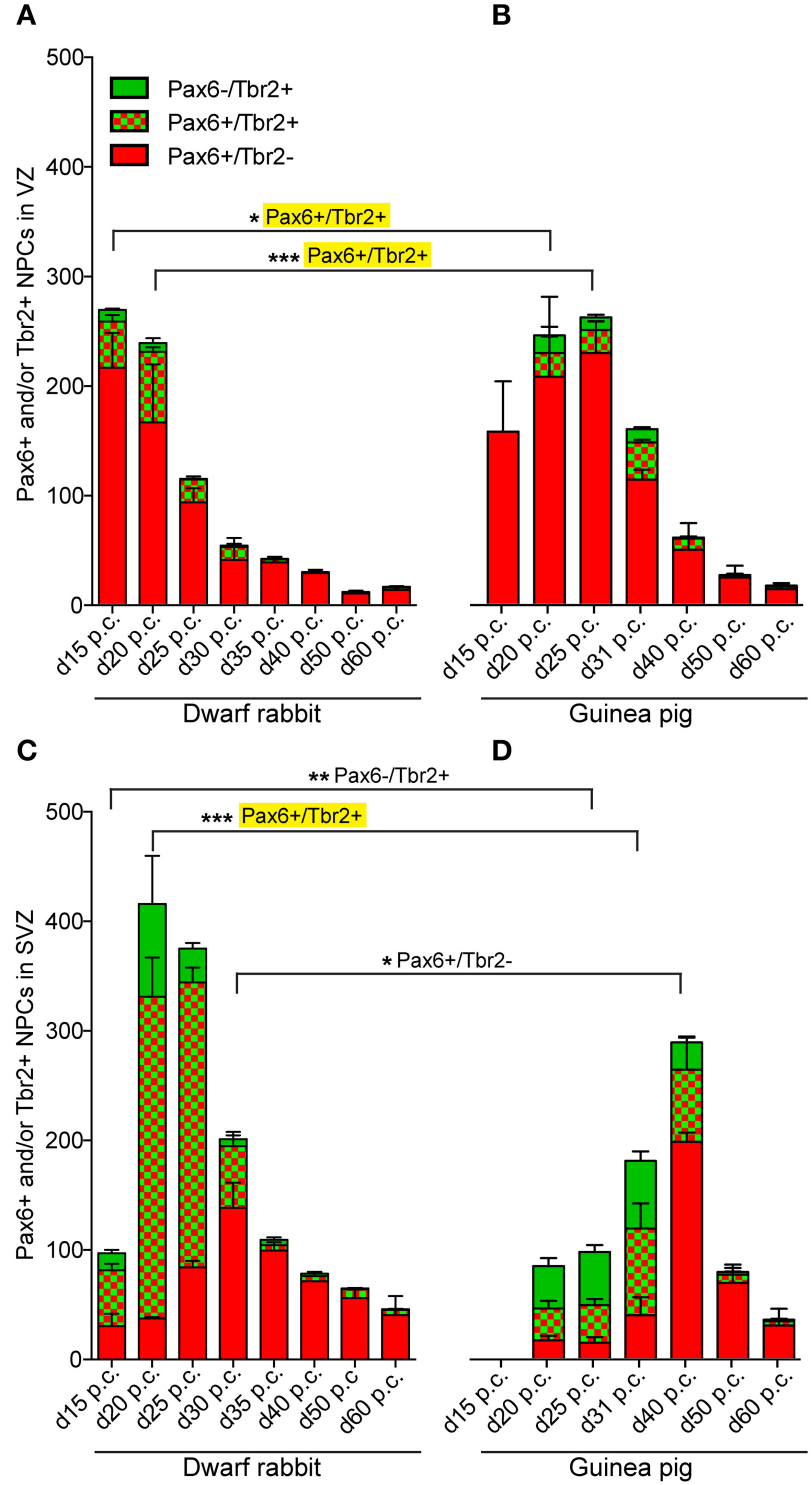
Quantification of Pax6+/Tbr2–, Pax6+/Tbr2+, and Pax6–/Tbr2+ NPCs in the VZ and SVZ of the developing guinea pig and dwarf rabbit neocortex. **(A–D)** Pax6+/Tbr2– (red), Pax6+/Tbr2+ (red/green), and Pax6–/Tbr2+ (green) NPCs in the VZ **(A,B)** and SVZ **(C,D)** of day 15 to 60 post-conception (p.c.) dwarf rabbit **(A,C)** and guinea pig **(B,D)** neocortex, expressed as number of cells per 100 μm ventricular surface. Y-axis for **(B)** is shown in **(A)**, y-axis for **(D)** is shown in **(C)**. Color legend is shown in **(A)**. For dwarf rabbits, the cortical wall corresponding to a total ventricular surface of 4.56–8.26 μm was analyzed. Data represent mean ± SD and are from two (d30 p.c., d35 p.c., d40 p.c., d50 p.c., d60 p.c.) or three (d15 p.c., d20 p.c., d25 p.c.) brains each. The cortical wall corresponding to a total ventricular surface of 2.70–13.68 μm was analyzed for the guinea pig. Data represent mean ± SD and are from two (d15 p.c., d20 p.c., d25 p.c.) or three (d31 p.c., d40 p.c., d50 p.c., d60 p.c.) brains each. Asterisks indicate statistically significant differences in Pax6+/Tbr2+ NPCs, Pax6+/Tbr2–, and Pax6–/Tbr2+ NPCs between corresponding neurogenesis stages of the dwarf rabbit and guinea pig, ***p < 0.001; **p < 0.01; *p < 0.05. Corresponding cortical neurogenesis stages were determined according to Workman et al. (2013) (www.translatingtime.org). For details, see the Materials and Methods section. Cell counts on a yellow background are significantly higher in the dwarf rabbit when compared with the guinea pig.

**Figure 4:**
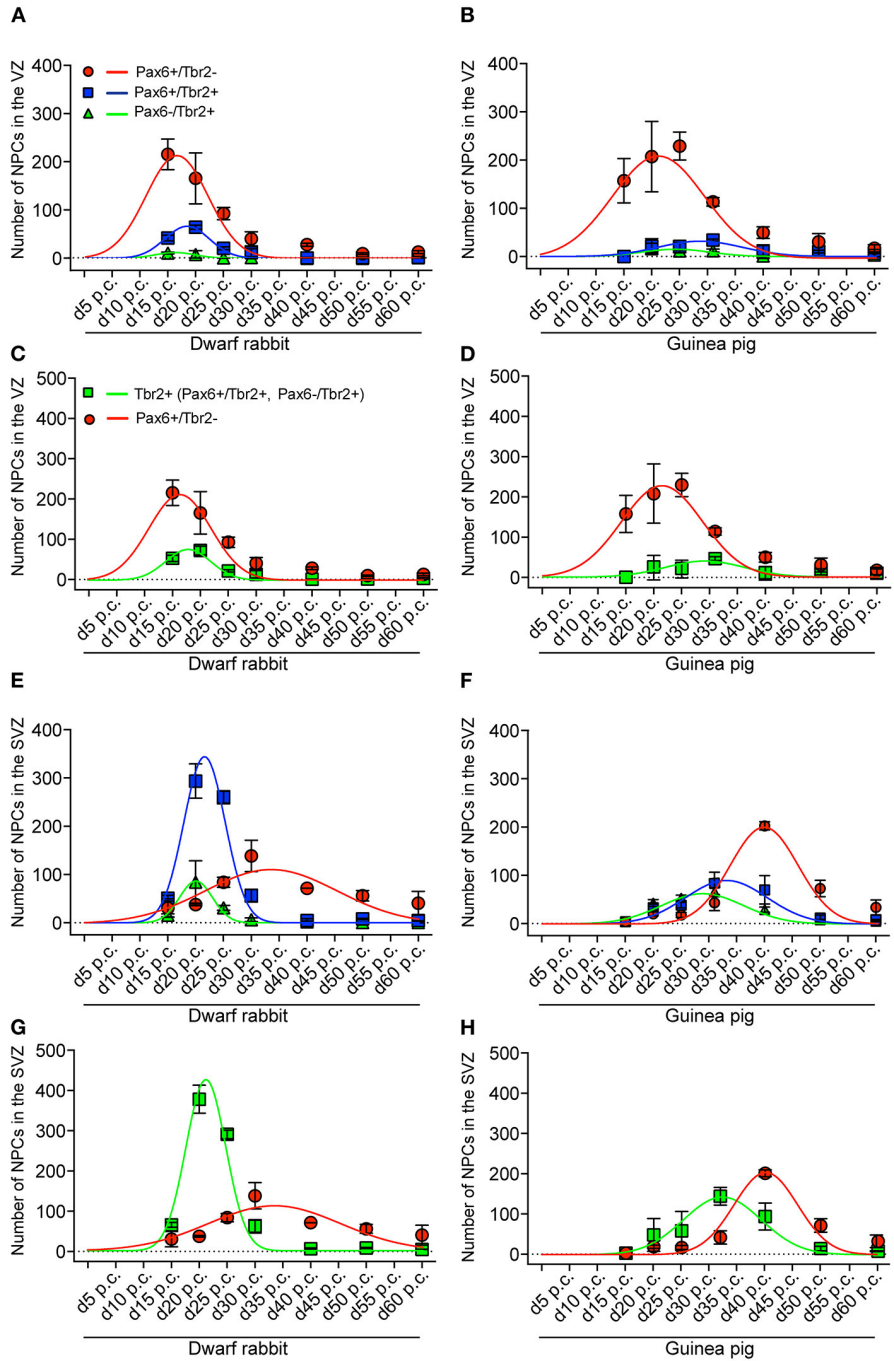
Extrapolation of NPCs abundance in the VZ and SVZ of the developing guinea pig and dwarf rabbit neocortex. **(A,B)** Pax6+/Tbr2– (red circles), Pax6–/Tbr2+ (green triangles), and Pax6+/Tbr2+ (blue rectangles) NPCs in the VZ of day 15 to 60 post conception (p.c.) dwarf rabbit and guinea pig neocortex, expressed as number of cells per 100 μm ventricular surface. Color legend is shown in **(A)**. **(C,D)** Pax6+/Tbr2– (red circles) and Tbr2+ (Pax6–/Tbr2+, Pax6+/Tbr2+, green rectangles) NPCs in the VZ of day 15 to 60 post conception (p.c.) dwarf rabbit and guinea pig neocortex, expressed as number of cells per 100 μm ventricular surface. Color legend is shown in **(C)**. **(E,F)** Pax6+/Tbr2– (red circles), Pax6–/Tbr2+ (green triangles), and Pax6+/Tbr2+ (blue rectangles) NPCs in the SVZ of day 15 to 60 post conception (p.c.) dwarf rabbit and guinea pig neocortex, expressed as number of cells per 100 μm ventricular surface. Color legend is shown in **(A)**. **(G,H)** Pax6+/Tbr2– (red circles) and Tbr2+ (Pax6–/Tbr2+, Pax6+/Tbr2+, green rectangles) NPCs in the SVZ of day 15 to 60 post conception (p.c.) dwarf rabbit and guinea pig neocortex, expressed as number of cells per 100 μm ventricular surface. Color legend is shown in **(C)**. **(A–H)** Data were obtained as in Figure 3. Development of NPCs in the VZ and SVZ was extrapolated based on Gaussian distribution. For details, see Materials and Methods section. **(A)** red line, *R*^2^ = 0.8964; green line, *R*^2^ = 0.6911; blue line, *R*^2^ = 0.9572; **(B)** red line, *R*^2^ = 0.8849; green line, *R*^2^ = 0.6944; blue line, *R*^2^ = 0.7487; **(C)** red line, *R*^2^ = 0.8964; green line, *R*^2^ = 0.9424; **(D)** red line, *R*^2^ = 0.8893; green line, *R*^2^ = 0.5212; **(E)** red line, *R*^2^ = 0.5694; green line, *R*^2^ = 0.7891; blue line, *R*^2^ = 0.9844; **(F)** red line, *R*^2^ = 0.9426; green line, *R*^2^ = 0.9190; blue line, *R*^2^ = 0.8249; **(G)** red line, *R*^2^ = 0.5694; green line, *R*^2^ = 0.9868; **(H)** red line, *R*^2^ = 0.9398; green line, *R*^2^ = 0.7716.

We first concentrated on APs as the primary NPCs. As observed in most mammalian species (Götz et al., 1998; Englund et al., 2005; Osumi et al., 2008; Fietz et al., 2010; Hansen et al., 2010; Reillo et al., 2011; Romer et al., 2018; Sauerland et al., 2018), NPCs of the dwarf rabbit and guinea pig VZ were Pax6+ and largely Tbr2– (Figures 1, 2, 3A,B). The highest abundance of Pax6+ NPCs in the VZ of the dwarf rabbit was observed at day 15 p.c. (Figures 1A, 3A). Indeed, extrapolation of the Pax6+ NPCs abundance in the dwarf rabbit VZ during early embryonic development revealed their maximum value to be generated around day 15 p.c. (Figures 4A,C). At day 15 p.c., the guinea pig VZ constitutes a very thin layer indicating that the generation of APs in the guinea pig neocortex might begin at a later postconceptional day when compared with that of the dwarf rabbit (Figure 2A). Similarly, the highest abundance of Pax6+ NPCs in the guinea pig VZ was detected at an important later time point after conception, i.e., at day 25 p.c. (Figures 2C, 3B). Interestingly, this shift in development has, if at all, only little impact on the maximum number of VZ NPCs generated per stage, as this number appears to be largely the same between the dwarf rabbit and guinea pig (Figures 3A,B). Once their maximum value is generated, the number of Pax6+ APs progressively declines in the further time course in both species analyzed (Figures 3A,B).

**Figure 5:**
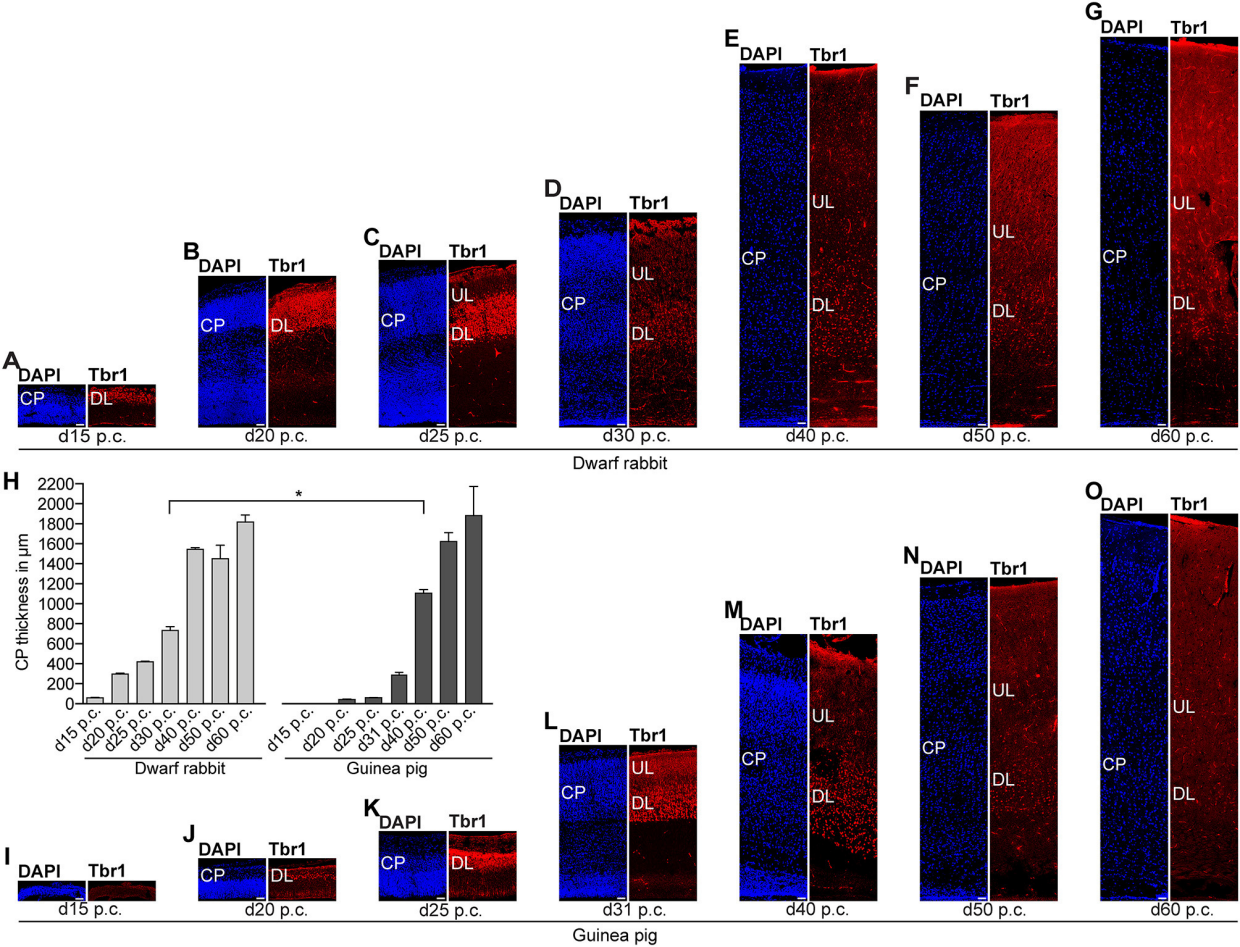
Tbr1 expression in the developing dwarf rabbit and guinea pig neocortex. **(A–G)** Immunofluorescence for Tbr1 (red) and DAPI staining (blue) on 30-μm cryosections of day 15 to 60 post-conception (p.c.) dwarf rabbit neocortex. **(H)** Quantification of CP thickness of d15–60 p.c. dwarf rabbit (light gray) and guinea pig (dark gray) neocortex. Data represent mean ± SD and are from two brains each. The asterisk indicates a statistically significant difference in CP thickness between corresponding neurogenesis stages of the dwarf rabbit and guinea pig, *p < 0.05. **(I–O)** Immunofluorescence for Tbr1 (red) and DAPI staining (blue) on 30-μm cryosections of day 15–60 p.c. guinea pig neocortex. **(A–G,I–O)** Scale bars, 50 μm. CP, cortical plate. DL, deep layer. UL, upper layer.

We next focused our analysis on BPs, which generate the majority of neurons in the developing neocortex (Haubensak et al., 2004; Miyata et al., 2004; Noctor et al., 2004; Hansen et al., 2010; Fietz and Huttner, 2011; Lui et al., 2011; Betizeau et al., 2013). To define the timing of the main period of cortical neurogenesis, we exploited previous findings that Tbr2+ NPCs are committed to neuronal fate and Tbr2 expression in progenitor compartments rises and falls with cortical plate neurogenesis (Englund et al., 2005; Hevner, 2019) and used the occurrence and abundance of Tbr2+ NPCs as a proxy for the estimation of the onset and end of cortical neurogenesis. The first occurrence of Tbr2+ NPCs in the guinea pig SVZ was observed at day 20 p.c. (Figure 2B). This coincides with the first observation of deep layers, mainly containing Tbr1+ neurons, in the guinea pig neocortex (Figures 5H,J). In contrast, in the dwarf rabbit neocortex, Tbr2+ NPCs—together with Tbr1+ neurons—are already present at day 15 p.c. (Figures 1A, 5A,H). Extrapolation of the Tbr2+ NPCs abundance in the dwarf rabbit germinal zones during early embryonic development revealed that they arise immediately before, i.e., at day 10 p.c. (Figures 4A,C,E,G). This indicates that the period of cortical neurogenesis starts at an earlier postconceptional day in the dwarf rabbit (day ~10 p.c.) compared with the guinea pig (day ~20 p.c.). The highest abundance of Tbr2+ NPCs (Figures 3C,D) was detected around the onset of the formation of upper layers, which mainly contain Tbr1– neurons (Figure 5), in the neocortex of both species analyzed. After their peak, the number of Tbr2+ NPCs decreases to minimal detectable levels until day 35 p.c. in the dwarf rabbit neocortex, indicating that neurogenesis is likely to end between day 30 and 35 p.c. (Figures 1E, 3C). In the guinea pig neocortex, the number of Tbr2+ NPCs reaches minimum values, not before day 50 p.c., suggesting neurogenesis to end between day 40 and 50 p.c. (Figures 2F, 3D), which is slightly later when compared with a previous study (Hatakeyama et al., 2017). Extrapolation of the Tbr2+ NPCs abundance in the SVZ of both species revealed their minimum value to be generated around 30 p.c. in the dwarf rabbit and 48 p.c. in the guinea pig (Figures 4G,H). Together, this suggests that the total length of the cortical neurogenic period is longer in the guinea pig (~28 d) than in the dwarf rabbit (~20 days).

**Figure 6:**
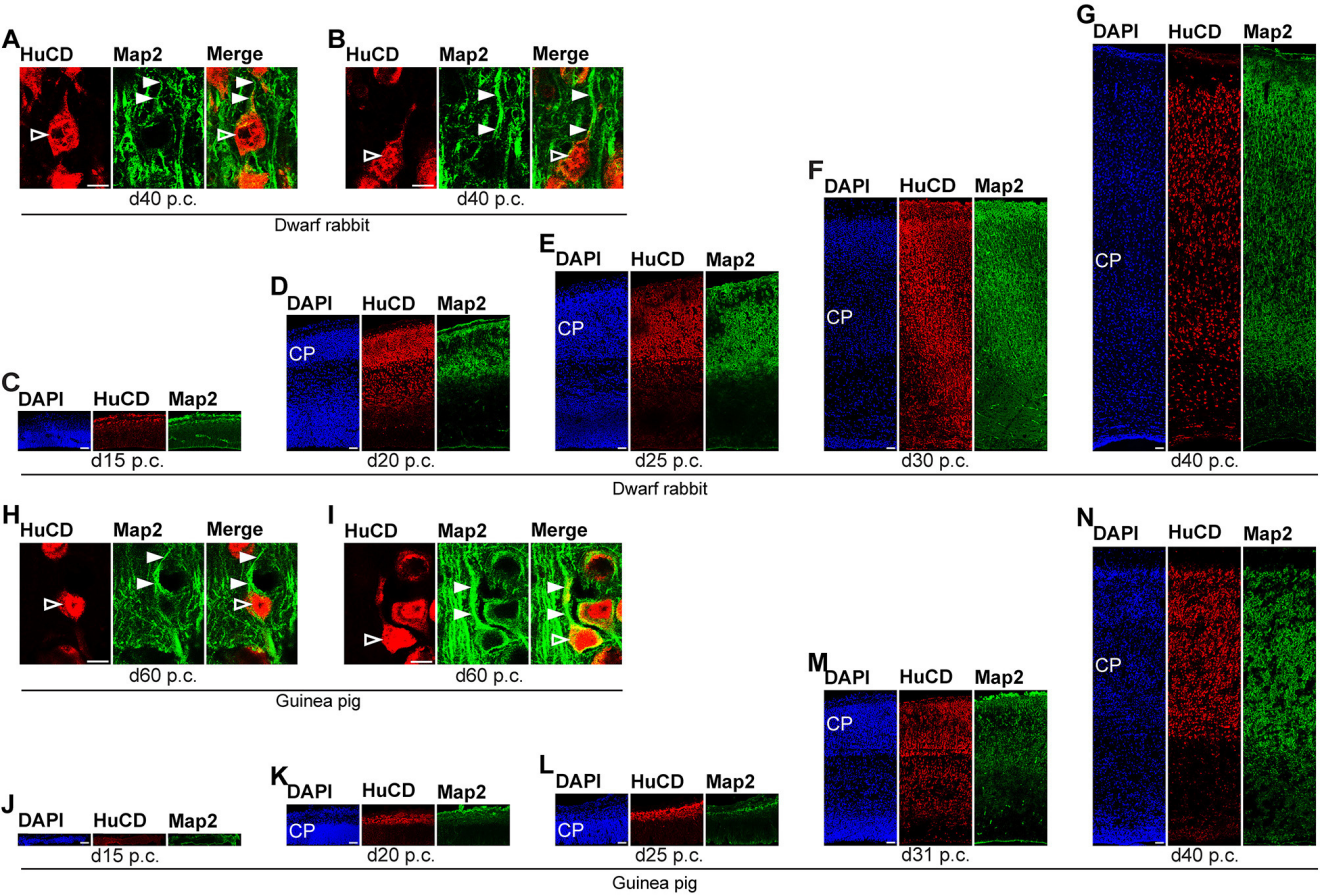
Hu C/D and Map2 expression in the developing dwarf rabbit and guinea pig neocortex. **(A–N)** Double immunofluorescence for Hu C/D (red, **A–N**) and Map2 (green, **A–N**) and DAPI staining (blue, **C–G,J–N**) on 30-μm cryosections of day 15 to 40 post-conception (p.c.) dwarf rabbit **(A–G)** and guinea pig **(J–N)** neocortex. Images in **(A,B,H,I)** show neurons with Hu C/D+ soma (open arrowhead) and extending Map2+ dendrites (solid arrowhead) in higher magnification of d40 p.c. dwarf rabbit **(A,B)** neocortex and d60 p.c. guinea pig **(H,I)** neocortex. Merge images in **(A,B,H,I)** show combined immunofluorescence of Hu C/D and Map2. CP, cortical plate. Scale bars, 10 μm **(A,B,H,I)** or 50 μm **(C–G,J–N)**.

**Figure 7:**
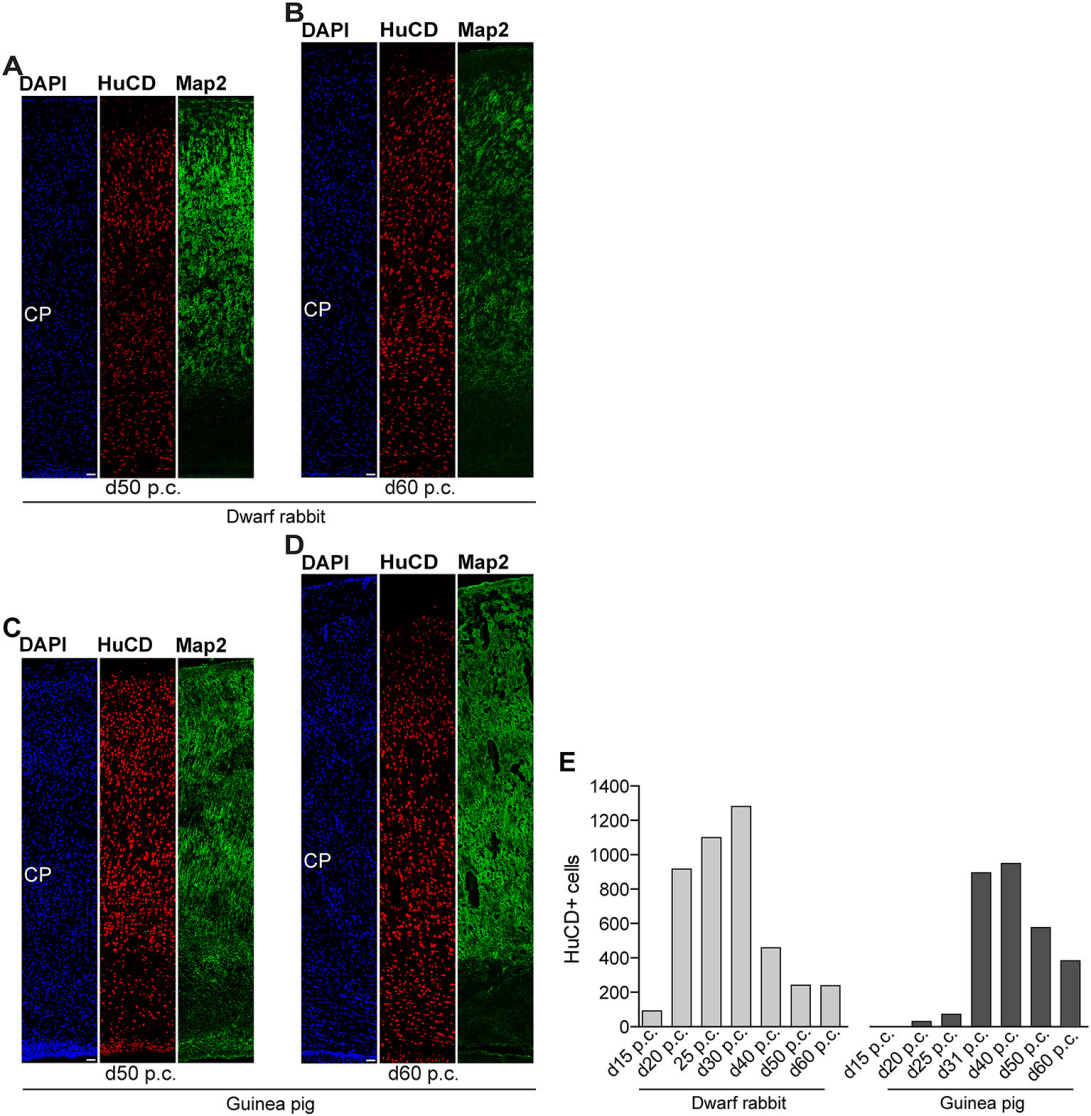
Hu C/D and Map2 expression in the developing dwarf rabbit and guinea pig neocortex. **(A–D)** Double immunofluorescence for Hu C/D (red) and Map2 (green) and DAPI staining (blue) on 30-μm cryosections of day 50 and 60 post-conception (p.c.) dwarf rabbit **(A,B)** and guinea pig **(C,D)** neocortex. Scale bars, 50 μm. CP, cortical plate. **(E)** Quantification of Hu C/D+ cells in d15–50 p.c. dwarf rabbit (light gray) and d20–60 p.c. guinea pig (dark gray) cortical wall, expressed as the number of cells per 100 μm ventricular surface. The cortical wall corresponding to a total ventricular surface of 3.86–6.02 μm (guinea pig) and 3.08–6.05 μm (dwarf rabbit) was analyzed. Data are from one brain each.

Interestingly, we observed a marked difference in the BP subtype composition between the developing dwarf rabbit and guinea pig neocortex during the respective main period of neurogenesis (Figures 3C,D). Specifically, the proportion of Tbr2+ NPCs that also express Pax6 is significantly higher in the dwarf rabbit SVZ than in the guinea pig SVZ when corresponding cortical neurogenesis stages, i.e., dwarf rabbit d20 and guinea pig d31 were compared (Figures 3C,D). Similarly, the number of Pax6+/Tbr2+ NPCs in the VZ, which resemble newborn BPs (Hevner, 2019), is significantly higher in the dwarf rabbit at day 15 and 20 p.c. when compared with that in the guinea pig SVZ at day 25 and 31 p.c., respectively (Figures 3A,B). Moreover, the maximum number of SVZ NPCs, i.e., the sum of Pax6+/Tbr2–, Pax6+/Tbr2+, Pax6–/Tbr2+ SVZ NPCs, generated at the respective peak stages of neurogenesis is higher in the dwarf rabbit (i.e., at day 20 p.c.) when compared with that of the guinea pig (i.e., at day 31 p.c.) (Figures 3C,D). Given that a higher abundance of NPCs generated would result in higher neuronal output (Rakic, 2009; Fietz and Huttner, 2011; Florio and Huttner, 2014; Dehay et al., 2015; Cardenas and Borrell, 2020), we found the number of neurons, identified by immunofluorescence for the pan-neuronal marker Hu C/D (Figures 6, 7) (Barami et al., 1995; Gao and Keene, 1996; Okano and Darnell, 1997; Wakamatsu and Weston, 1997), generated at the respective end of cortical neurogenesis to be higher in the developing wall of the dwarf rabbit (i.e., at day 30 p.c.) when compared with that of the guinea pig (i.e., at day 50 p.c.) (Figure 7E). To investigate whether these developmental differences impact adult brain size, we obtained the absolute brain mass. We calculated the encephalization quotient as a between-species measure for relative brain mass for both species. Indeed, this revealed that the dwarf rabbit is characterized by a higher absolute brain mass and exhibits a higher encephalization quotient when compared with that of the guinea pig (Table 1).

**Table 1:** Adult brain mass, EQ, and IND of the dwarf rabbit and guinea pig.

**Species**	**Adult brain mass (g)**	**EQ**	**IND (%)**	
			**Day 30 p.c**.	**Day 60 p.c**.
Dwarf rabbit	9.6	0.72	10.53 (time of birth)	60.88
Guinea pig	4.3	0.46	4.85	67.82 (time of birth)

*The respective time of birth is indicated in brackets. For details, see the Materials and Methods section. EQ, encephalization quotient; IND, index of neural development; p.c., post-conception*.

### Developmental Differences in Gliogenesis and Neuron Maturation Between the Dwarf Rabbit and Guinea Pig Neocortex

**Figure 8:**
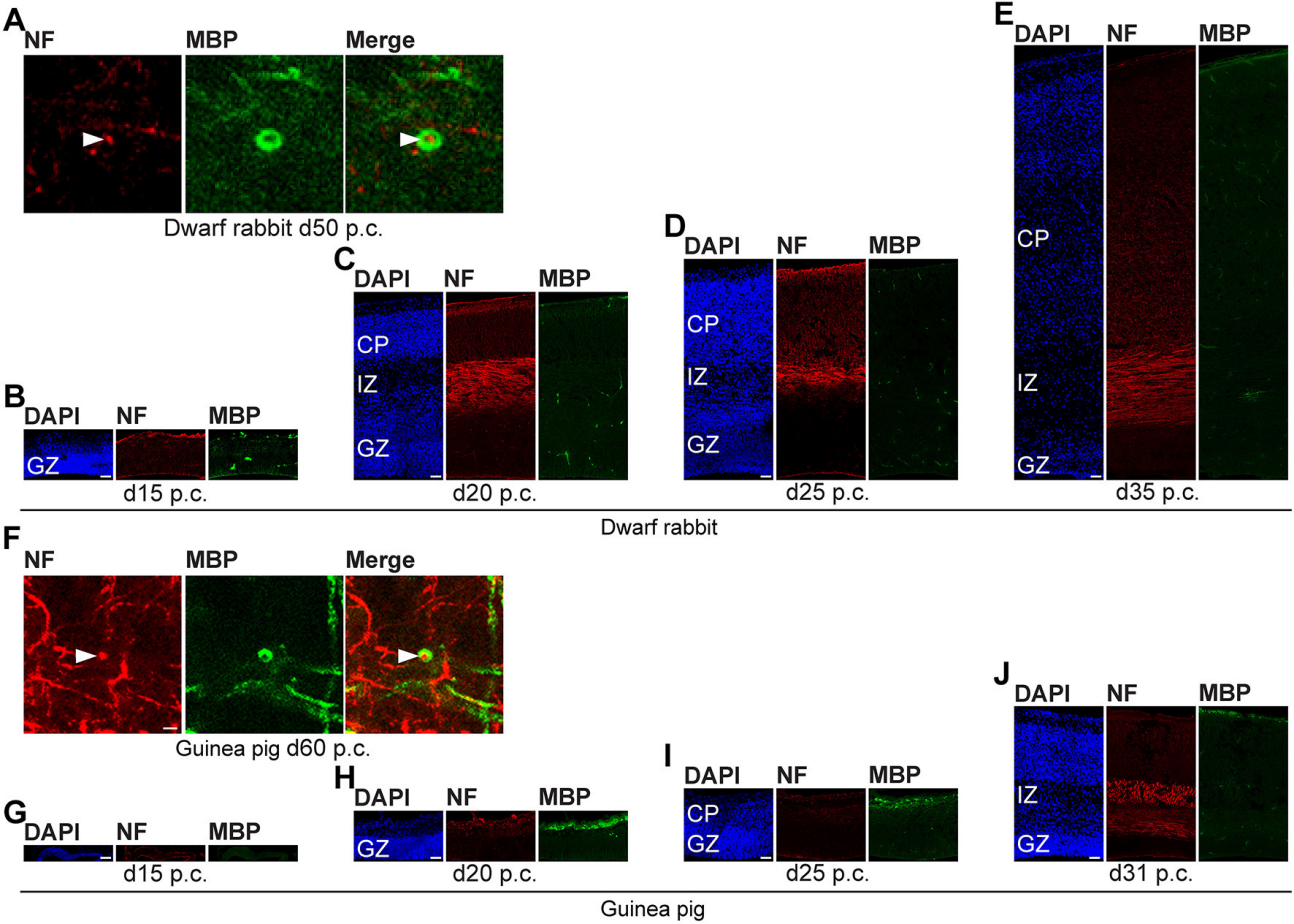
Neurofilament H and MBP expression in the developing dwarf rabbit and guinea pig neocortex. **(A–J)** Double immunofluorescence for neurofilament H (NF, red, **A–J**) and MBP (green, **A–J**) and DAPI staining (blue, **B–E,G–J**) on 30-μm cryosections of day 15–35 post-conception (p.c.) dwarf rabbit neocortex and d15–31 p.c. guinea pig neocortex. Images in **(A,F)** show NF+ extension (solid arrowhead) surrounded by MBP+ myelin sheath in higher magnification of d50 p.c. dwarf rabbit neocortex **(A)** and d60 p.c. guinea pig neocortex **(F)**. Merge images in **(A,F)** show combined immunofluorescence of NF and MBP. CP, cortical plate. GZ, germinal zone. IZ, intermediate zone. Scale bars, 2 μm **(A,F)** or 50 μm **(B–E,G–J)**.

**Figure 9:**
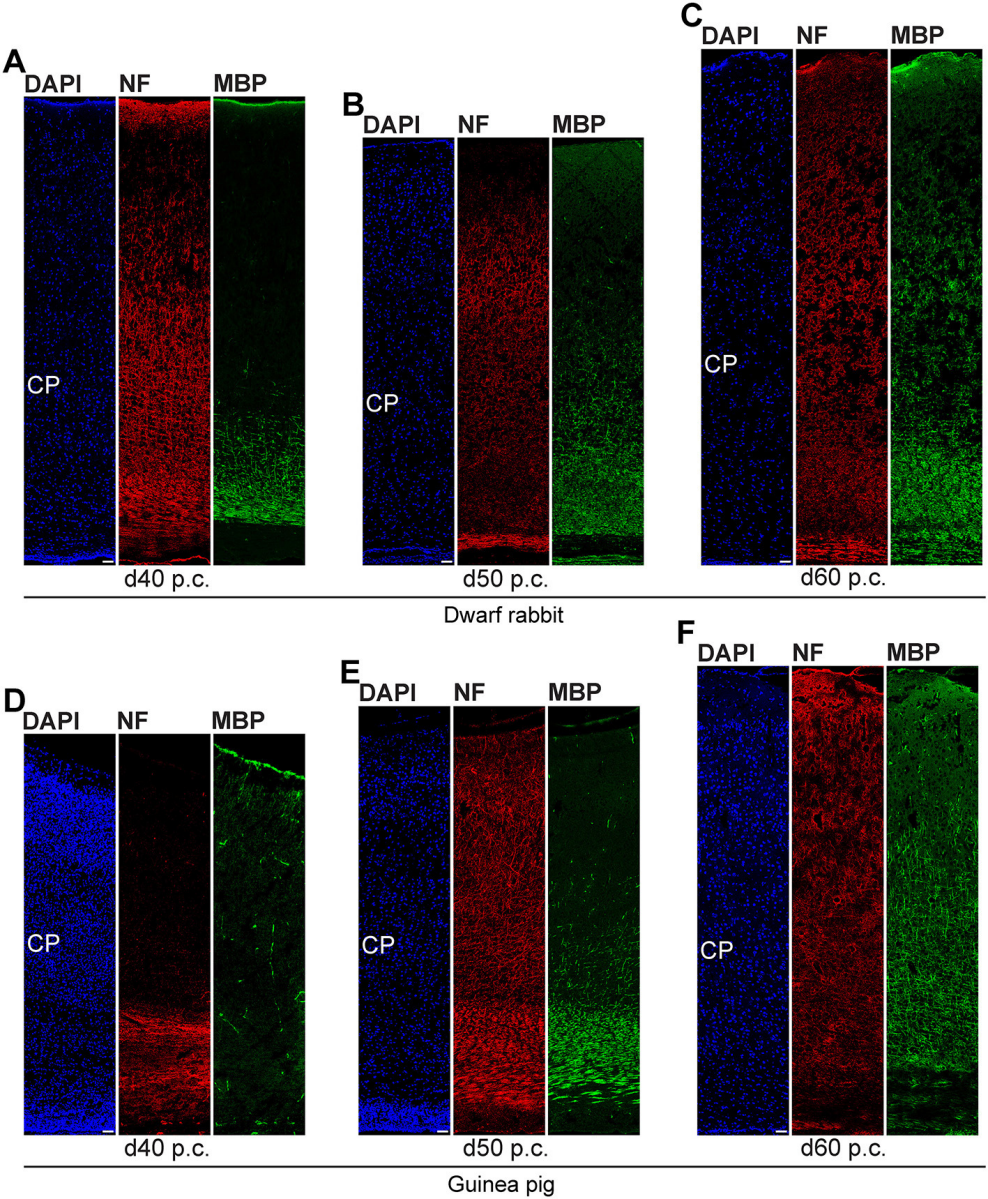
Neurofilament H and MBP expression in the developing dwarf rabbit and guinea pig neocortex. **(A–F)** Double immunofluorescence for neurofilament H (NF, red) and MBP (green) and DAPI staining (blue) on 30-μm cryosections of day 40–60 post-conception (p.c.) dwarf rabbit **(A–C)** and guinea pig **(D–F)** neocortex. CP, cortical plate. Scale bars, 50 μm.

In the next step, we focused our analysis on the spatial and temporal characterization of distinct parameters of neuron maturation. We analyzed cortical sections of the dwarf rabbit and guinea pig by double-immunofluorescence for Hu C/D and Map2, a marker for neuronal dendrites (Figures 6, 7) (Bernhardt and Matus, 1984; Chen et al., 1992; Dehmelt and Halpain, 2005). In both species analyzed, Map2+ structures extend from and partly localize with Hu C/D+ somata (Figures 6A,B,H,I) and were mainly detected in the developing CP (Figures 6, 7). Their first appearance coincides with the first detection of neurons in the developing dwarf rabbit and guinea pig neocortex, which is in line with the notion that dendrite formation constitutes one of the initial steps in the process of neuron maturation (Bernhardt and Matus, 1984; Bernhardt et al., 1985; Marin-Padilla, 1992; Whitford et al., 2002; Jan and Jan, 2003). The process of axon formation was examined by double-immunofluorescence for the axonal marker neurofilament H (Figures 8, 9) (Shaw and Weber, 1982; Carden et al., 1987; Lariviere and Julien, 2004; Lyck et al., 2008) and myelin basic protein (MBP), a marker for myelination in the central nervous system (Foran and Peterson, 1992; Zecevic et al., 1998; Lyck et al., 2008). In both species analyzed, an intense and widespread appearance of axonal structures was first detected in the intermediate zone (IZ) ~10 days after the respective onset of cortical neurogenesis; i.e., at day 20 p.c. in the dwarf rabbit and day 31 p.c. in the guinea pig (Figures 8C,J). Intriguingly, these axonal extensions were mostly oriented parallel to the ventricular surface (Figures 8C–E,J, 9D), thus, supporting previous studies that have identified the IZ to be populated by interneurons that migrate tangentially from the ventral into the dorsal telencephalon during early embryonic development (DeDiego et al., 1994; De Carlos et al., 1996; Lavdas et al., 1999; Letinic et al., 2002; Marin and Rubenstein, 2003; Wonders and Anderson, 2006; Hansen et al., 2013). In the CP, axonal processes, which were mainly oriented radially to the ventricular surface, were already detected as early as day 20 p.c. in the dwarf rabbit (Figure 8C) and day 31 p.c. in the guinea pig (Figure 8J); however, they only become numerous with day 40 p.c. in the dwarf rabbit (Figure 9A) and day 50 p.c. in the guinea pig (Figure 9E), and thus after the end of the main period of cortical neurogenesis. Similarly, widespread myelination in the CP was not detected until day 40 p.c. in the dwarf rabbit (Figure 9A) and day 50 p.c. in the guinea pig (Figure 9E). Once axonal processes are generated in high abundance, we observed a widespread formation of synapses, as identified by the presynaptic marker synaptophysin (Wiedenmann and Franke, 1985; Gil-Loyzaga and Pujol, 1988; Ichikawa et al., 1991), in the day 40 p.c. dwarf rabbit and day 50 p.c. guinea pig CP (Supplementary Figure 3).

**Figure 10:**
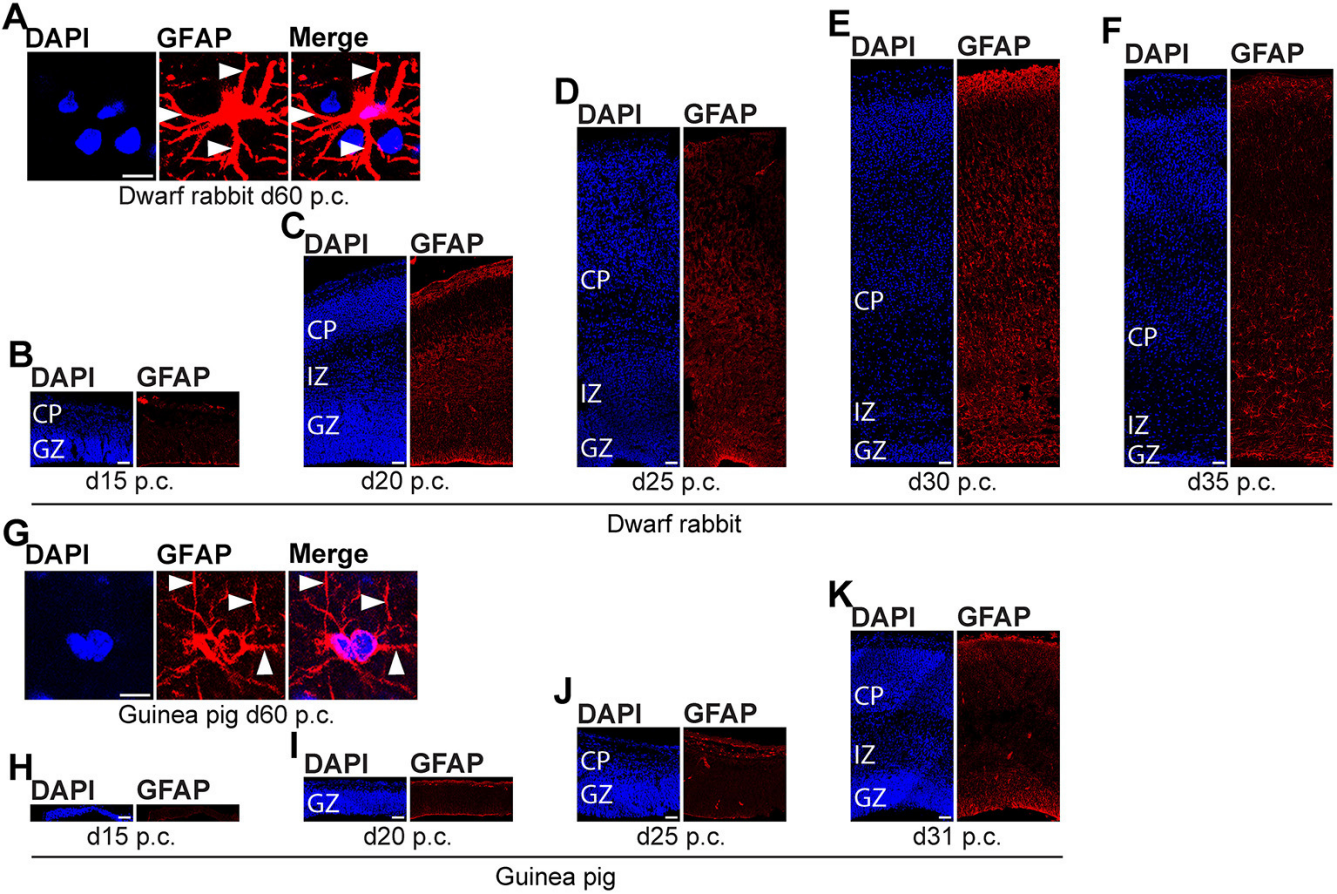
GFAP expression in the developing dwarf rabbit neocortex and guinea pig neocortex. **(A–K)** Immunofluorescence for GFAP (red) and DAPI staining (blue) on 30-μm cryosections of day 15–35 post-conception (p.c.) dwarf rabbit **(A–F)** and d15-31 p.c. guinea pig **(G–K)** neocortex. Images in **(A,G)** show GFAP+ astrocyte with soma and extending processes (solid arrowhead) in higher magnification of d60 p.c. dwarf rabbit **(A)** and d60 p.c. guinea pig **(F)** neocortex. Merge images in **(A,G)** show combined fluorescence of GFAP and DAPI. CP, cortical plate. GZ, germinal zone. IZ, intermediate zone. Scale bars, 10 μm **(A,G)** and 50 μm **(B–F,H–K)**.

**Figure 11:**
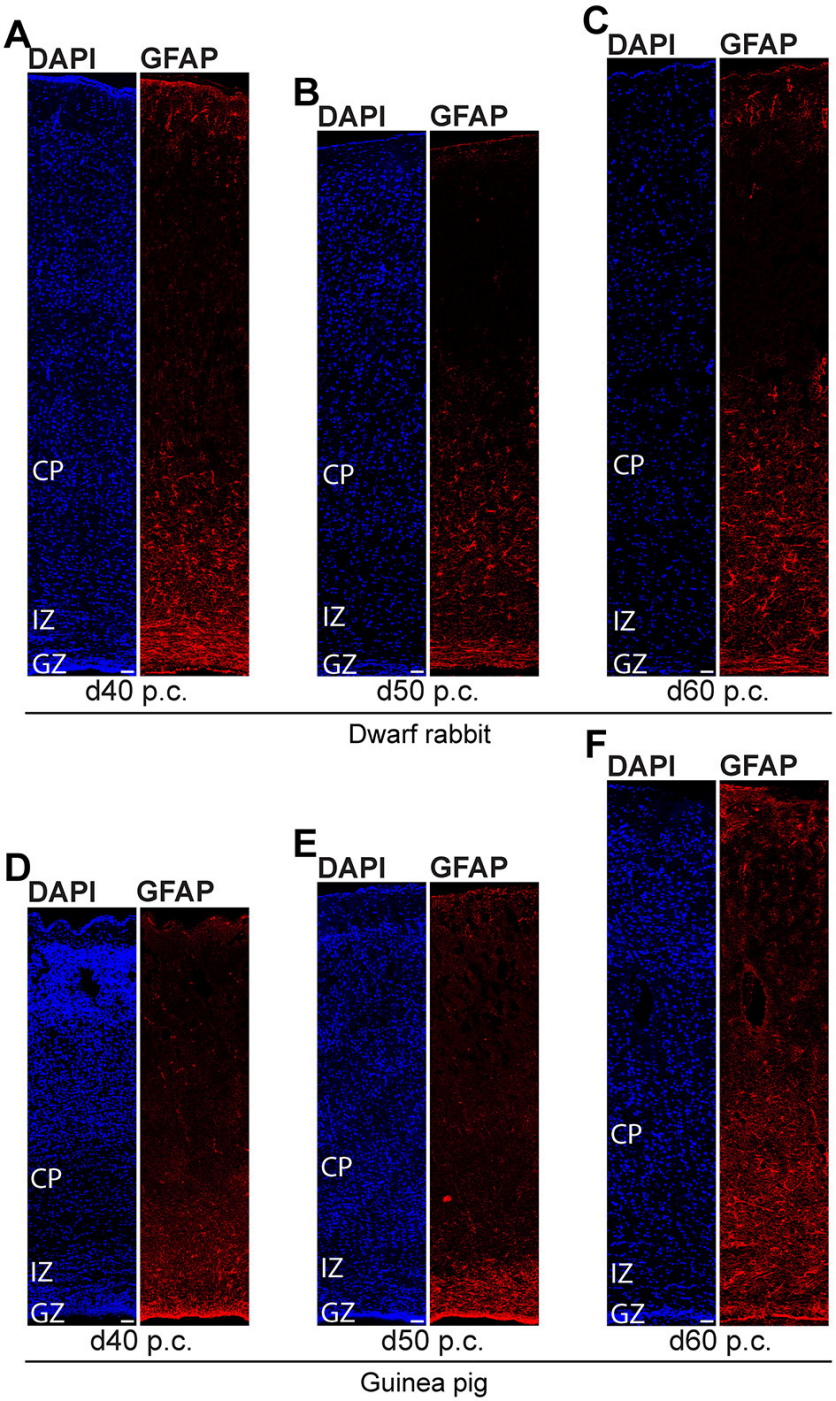
GFAP expression in the developing dwarf rabbit neocortex and guinea pig neocortex. Immunofluorescence for GFAP (red) and DAPI staining (blue) on 30-μm cryosections of day 40–60 post-conception (d p.c.) dwarf rabbit **(A–C)** neocortex and guinea pig **(D–F)** neocortex. CP, cortical plate. GZ, germinal zone. IZ, intermediate zone. Scale bars, 50 μm.

We further investigated the formation of astrocytes using immunohistochemistry for GFAP that resembles the hallmark intermediate filament protein in astrocytes (Figures 10, 11) (Bignami and Dahl, 1973; Kalman and Hajos, 1989; Eng et al., 2000; Hol and Pekny, 2015). In both species analyzed, GFAP+ structures were already detected during mid-neurogenesis, i.e., at day 20 p.c. in the dwarf rabbit neocortex and day 31 p.c. in the guinea pig neocortex (Figures 10C,K). As GFAP expression was mainly observed in the germinal zones close to the lateral ventricle (Figures 10C,K), our data suggest GFAP be expressed by distinct NPCs, e.g., radial glial cells, in the developing dwarf rabbit and guinea pig neocortex, which is in line with observations in other mammalian species (Noctor et al., 2002; Kriegstein and Alvarez-Buylla, 2009; Fietz et al., 2010, 2020; Kelava et al., 2012). Mature GFAP+ astrocytes, which exhibit a typical star-shaped appearance (Figures 10A,G), were first detected at high numbers at day 40 p.c., in the dwarf rabbit (Figure 11A) and day 50 p.c., in the guinea pig neocortex (Figure 11E). Again, this suggests that major aspects of astrocyte formation occur after the end of the main period of cortical neurogenesis in both species analyzed.

**Figure 12:**
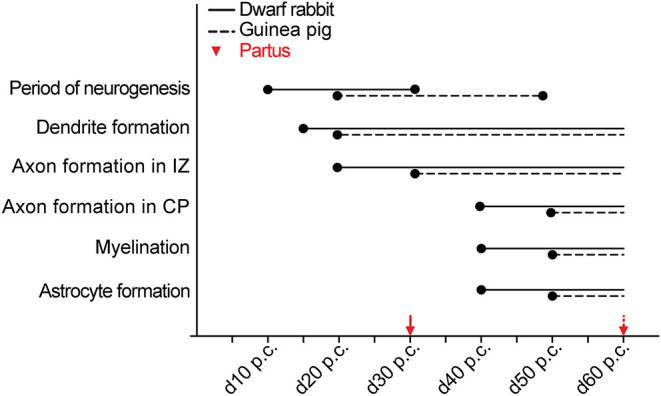
Comparison of specific neurodevelopmental events between the dwarf rabbit and guinea pig neocortex. Onset and duration of the period of neurogenesis, dendrite formation, axon formation, myelination, and astrocyte formation in the dwarf rabbit and guinea pig neocortex between day 10 and 60 post-conception (p.c.). Data for the period of neurogenesis are based on the development of Tbr2+ NPCs in the SVZ (Figure 4); data for dendrite formation, axon formation, myelination, and astrocyte formation are based on immunofluorescence staining (Figures 6–11). For details, see the Results section. Data for dwarf rabbits are shown in solid black lines, data for guinea pigs are shown in black dashed lines. Red arrows indicate the respective time of birth (partus) of the dwarf rabbit (solid line) and guinea pig (dashed line). IZ, intermediate zone. CP, cortical plate.

Together, our data show that the basic order of neuro- and gliogenesis and neuron maturation is preserved in the dwarf rabbit and guinea pig neocortex. In absolute postconceptional days, the onset of cortical neurogenesis starts at a later time point in the guinea pig compared with the dwarf rabbit (Figure 12). However, when expressed concerning the gestation length, the onset of cortical neurogenesis occurs at a similar time point in both species analyzed, i.e., at 33% of gestation corresponding to the end of the first trimester of gestation. In both species, the main period of cortical neurogenesis ends before birth, lasting until the beginning of the third trimester in the guinea pig neocortex until the end of gestation in the dwarf rabbit neocortex (Figure 12). Concerning neuron maturation, dendrite formation tends to start during early-mid neurogenesis. In contrast, major aspects of axon formation and myelination together with astrogenesis seem to occur once neurogenesis is largely terminated in the neocortex of both species analyzed (Figure 12). Taken the time of birth into account, both species exhibit a different cortical growth and maturation status at birth. While the neocortex of the guinea pig contains neurons that seemingly exhibit well-developed dendrites and myelinated axons as well as astrocytes, the dwarf rabbit neocortex seems to lack neurons exhibiting well-developed and myelinated axons and astrocytes at the time of birth (Figure 12). This indicates that—in contrast to the guinea pig neocortex—a greater proportion of growth and maturation in the dwarf rabbit neocortex will occur during postnatal development. To test this, we calculated the index of neural development (IND) as the ratio between developmental and adult brain mass at the time of birth for both species analyzed. This shows that the guinea pig achieves an IND > 50% at the time of birth, and thus major brain growth in the guinea pig occurs during prenatal development. In contrast, in the dwarf rabbit exhibiting an IND of ~10% at the time of birth, the overwhelming majority of brain growth is achieved during postnatal development (Table 1). Interestingly, similar to the guinea pig, the dwarf rabbit achieves an IND > 50% at day 60 p.c. (Table 1). Moreover, at day 60 p.c., the dwarf rabbit neocortex is characterized by neurons with structurally well-developed and myelinated axons and astrocytes as has been observed for the guinea pig of the same postconceptional day (Figures 9C,F, 11C,F).

## Discussion

This study compares distinct patterns of neocortex development, specifically neurogenesis, gliogenesis, and neuron maturation, between the precocial guinea pig and the altricial dwarf rabbit. By using two mammalian species that show a relatively close phylogenetic and genetic relatedness and several similarities concerning adult and birth body weight and biology (e.g., food uptake and digestion, habitation and social behavior) (King, 1956; Kunkel and Kunkel, 1964; Sachser, 1998; Asher et al., 2004; McDougall et al., 2009; Thormann, 2012; Varga, 2014; Upham et al., 2019; Lundwall et al., 2020), we attempted to minimize the confounding factors that potentially affect brain development. Our data demonstrate that the basic order of distinct cortical neurodevelopmental events is preserved in the altricial dwarf rabbit and precocial guinea pig. Specifically, we found neurogenesis, e.g., including the sequential generation of DL and UL neurogenesis, to precede gliogenesis, as observed in the neocortex of other mammalian species (Lee et al., 2000; Sauvageot and Stiles, 2002; Kriegstein and Alvarez-Buylla, 2009). Moreover, we found the principal order of dendrite and axon formation and myelination to be similar between the altricial dwarf rabbit and precocial guinea pig neocortex.

Intriguingly, our data show that, in absolute postconceptional days, the onset of cortical neurogenesis is shifted between the two species analyzed, starting ~10 days later in the precocial guinea pig than in the altricial dwarf rabbit. This time shift corresponds to the translating time between equivalent post conception dates of rabbit and guinea pig (Workman et al., 2013; Finlay and Huang, 2020). Moreover, we show that the period of cortical neurogenesis takes longer in absolute gestational days in the precocial guinea pig compared with that of the altricial dwarf rabbit. Thus, our findings are in line with previous studies suggesting precocial mammals exhibit a delayed onset and protracted duration of cortical neurogenesis when compared with phylogenetically closely related altricial mammals (Brunjes et al., 1989; Brunjes, 1990; Workman et al., 2013; Finlay and Huang, 2020). In this regard, it is interesting to note that other neurodevelopmental processes, including the transformation of the neural plate into the neural tube as well as hippocampal neurogenesis, the generation of olfactory mitral cells, and that of retinal rods and cones seem to start at a later time point after conception and to last for a longer absolute duration in the precocial guinea pig when compared with the altricial rabbit (DeSesso, 1996; Schnorr and Kressin, 2006; Workman et al., 2013). Moreover, developmental milestones of the formation of primitive structures, including the primitive streak and somites, and other organs such as liver and lung, initiate at a substantially later time point after conception in the precocial guinea pig than in the altricial rabbit (DeSesso, 1996). Together this indicates that the time-shifted onset and prolonged period of development is not specific to the nervous system of the precocial guinea pig but instead a more common feature of its embryonic and fetal development involving many different tissues and organs. Given that precocial species, in general, are characterized by a longer gestation period than closely related altricial species, it is tempting to speculate that the onset and duration of distinct developmental processes, e.g., cortical neurogenesis, are primarily linked to gestation length (rather than life history patterns at birth, i.e., altriciality and precociality) (Dieterlen, 1963; Martin and Maclarnon, 1985; Derrickson, 1992; Finlay and Uchiyama, 2017; Scheiber et al., 2017). This is supported by our findings, showing that the onset of neurogenesis occurs at a similar time point, when expressed as percentage of gestation, i.e. at the end of the first trimester of gestation, in the dwarf rabbit and guinea pig neocortex. Further evidence for this comes from recent studies revealing the lengths of neurogenesis and gestation to be tightly related (Lewitus et al., 2014; Glatzle et al., 2017; Stepien et al., 2020). Moreover, previously published findings and unpublished data from our laboratory show that within the order of carnivores, consisting of species with similar life history patterns at birth, species exhibiting a longer gestation period, i.e., cat, seem to be characterized by later onset and more protracted period of cortical neurogenesis and other distinct neurodevelopmental processes when compared with species with shorter gestation period such as ferret (Fietz et al., 2010; Reillo et al., 2011; Reillo and Borrell, 2012; Workman et al., 2013). Understanding the factors responsible for these temporal differences and the potential mutual regulation of brain growth and that of other somatic organ systems during gestation would lend essential insights into the evolutionary mechanism involved in the process of speciation within the altricial–precocial spectrum. In this regard, it would be interesting to compare the neural developmental data obtained in the dwarf rabbit in this study to a precocial species of the same order, i.e., the European hare (*Lepus europaeus*).

Previous studies have linked a lengthening of the neurogenic period to higher neuron production and cortex expansion (Florio and Huttner, 2014; Lewitus et al., 2014; Cardenas and Borrell, 2020; Finlay and Huang, 2020; Stepien et al., 2020). However, our data reveal that the neuronal output generated during peak stages of cortical neurogenesis and the absolute and relative adult brain mass is lower in the guinea pig than in the dwarf rabbit. Interestingly, we found a marked difference in the BP subtype composition between the developing dwarf rabbit and guinea pig neocortex at peak stages of neurogenesis. Specifically, we found the proportion of Tbr2+ BPs that also express Pax6 to be markedly higher in the dwarf rabbit than in the guinea pig neocortex. On the assumption that sustainment of Pax6 expression in BPs is linked to higher cell proliferation (Betizeau et al., 2013; Wong et al., 2015), our data suggest that the developing dwarf rabbit neocortex contains a higher abundance of highly proliferative BPs, which might enable the dwarf rabbit to achieve a higher neuron production and brain size when compared with that of the guinea pig, thereby counterbalancing its shorter neurogenesis length. Indeed, the more rapid and higher expansion of the BP cell pool, which is not accompanied by an equivalent decrease in the AP cell number, supports the notion of self-amplifying NPCs being present in higher abundance in the dwarf rabbit SVZ when compared with that of the guinea pig. Together, our data provide evidence for the notion that neuron production in the developing neocortex is determined by complex regulation of multiple factors, including the duration of the neurogenesis period, the absolute number of NPCs, and the relative abundance of each NPC type (Florio and Huttner, 2014; Lewitus et al., 2014; Cardenas and Borrell, 2020). Therefore, further studies that use a larger number of animals and address the mode and rate of cell division of distinct NPC subtypes are important to directly evaluate their contribution to neocortex development in the dwarf rabbit and guinea pig.

Strikingly, we observed that the IND of the precocial guinea pig is considerably higher at the time of birth when compared with that of the altricial dwarf rabbit, which is in line with previously published data showing precocial species to give birth to larger-brained offspring after controlling for body size (Barton and Capellini, 2011). In this respect, our data indicate that the guinea pig neocortex exhibits a higher maturation status, containing more neurons with well-developed dendrites and myelinated axons and astrocytes, than the dwarf rabbit, in which major steps of neuron maturation, i.e., axon generation and myelination, and astrogenesis mainly set in after birth. Thus, our data provide evidence for the notion that brain expansion in the precocial newborn is largely due to prenatally initiating processes of gliogenesis and neuron maturation, instead of increased neurogenesis as previously discussed (Glatzle et al., 2017). Moreover, they are in line with previously published data showing that the precocial index, which characterizes the point of neural maturation at birth, is higher in the guinea pig (0.841) than in the rabbit (0.537) and confirm the expected contrast of the position of birth concerning specific neural milestones for the precocial vs. closely related altricial mammals (Workman et al., 2013; Finlay and Uchiyama, 2017). Given that the offspring of the precocial guinea pig, in contrast to that of the dwarf rabbit, is born with advanced cognitive, sensory, and locomotor abilities, our findings indicate that its offspring has acquired the morphological machinery required to attain its high functional state at birth. Interestingly, in the dwarf rabbit, the cortical maturation status and the ratio between developmental and adult brain mass largely catch up to those of the guinea pig during early postnatal development, achieving an IND > 50%, until day 60 p.c., a stage which marks the approximate end of weaning in the dwarf rabbit with its pups having open ears and eyes and being motorically competent (Varga, 2014). Further studies using an additional set of immunohistochemical markers of neuronal maturation as well as electrophysiological techniques, i.e., patch clamp, are needed to precisely evaluate the process of neuron and glia maturation and to demonstrate whether the existing neurons in the pre- and neonatal guinea pig and early postnatal dwarf rabbit neocortex are indeed genuine, and thus functional mature.

In conclusion, this study provides comprehensive data on distinct patterns of brain development between the precocial guinea pig and the altricial dwarf rabbit, which may serve as empirical reference data in future studies. While the basic order of cortical neuro- and gliogenesis and neuron maturation is the same during early development, their specific timing markedly differs concerning the postconceptional age and the time of birth between them. Moreover, our data provide evidence for the notion that a complex regulation of multiple factors determines the amount of neuron production in the developing neocortex of the guinea pig and dwarf rabbit. Together, these data expand our current understanding of the timing and cellular differences that regulate patterns of mammalian brain growth and maturation and provides a better understanding of the evolution of mammalian altriciality and presociality.

## Data Availability Statement

The raw data supporting the conclusions of this article will be made available by the authors, without undue reservation.

## Ethics Statement

The animal study was reviewed and approved by Landesdirektion Leipzig.

## Author Contributions

MK and SF conceived and designed the experiments and analyzed the data. MK, MH, and CS performed the experiments. MK, MM, and SF discussed the data and wrote the article. All authors reviewed and approved the manuscript.

## Conflict of Interest

The authors declare that the research was conducted in the absence of any commercial or financial relationships that could be construed as a potential conflict of interest.
