# Supplementary material for: Developmental Differences in Neocortex Neurogenesis and Maturation Between the Altricial Dwarf Rabbit and Precocial Guinea Pig
Source: Front Neuroanat. 2021 May 31;15:678385. doi: 10.3389/fnana.2021.678385 (PMC8200626; doi:10.3389/fnana.2021.678385)
Supplement: Supplementary file 1 [file Data_Sheet_1.PDF]

Supplementary Material

Developmental differences in neocortex neurogenesis and maturation between the altricial dwarf rabbit and precocial guinea pig

Mirjam Kalusa, Maren D. Heinrich, Christine Sauerland, Markus Morawski and Simone A. Fietz*

*Correspondence: simone.fietz@vetmed.uni-leipzig.de

Supplementary Figures

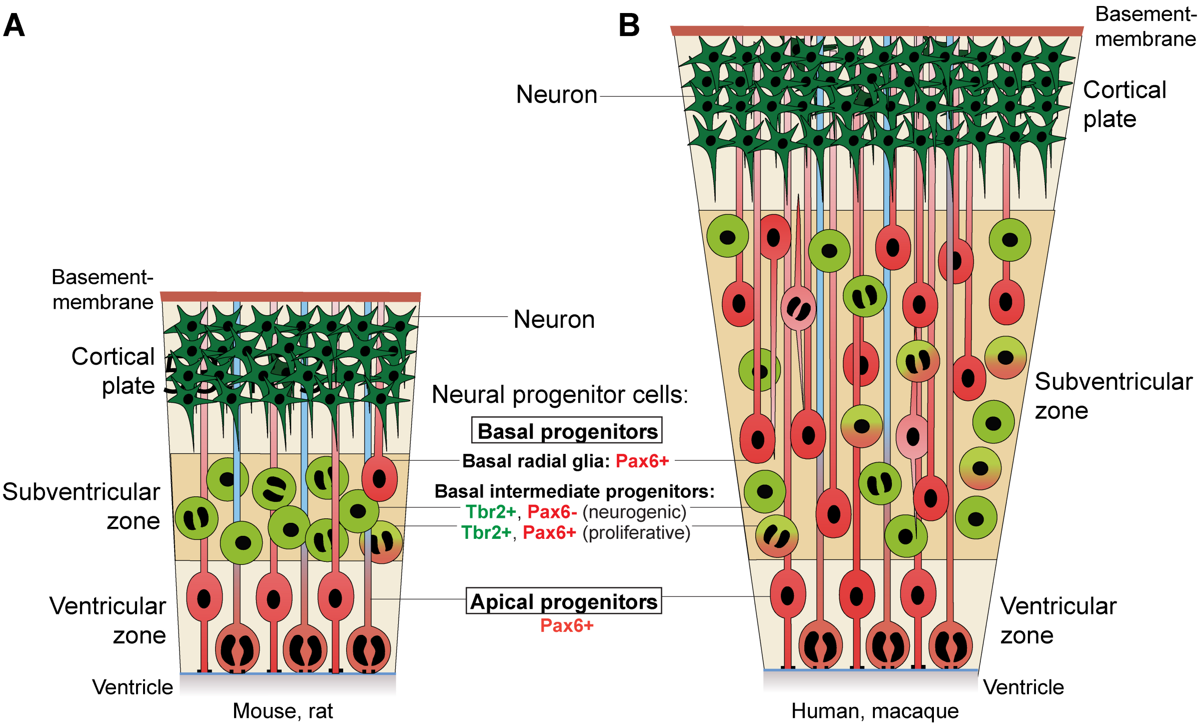

SUPPLEMENTARY FIGURE 1 | Overview of the major neural progenitor cell types in the mammalian neocortex. Schematic drawing of the location, marker expression and morphology of the major neural progenitor cell types in the developing rodent, i.e. mouse and rat (A) and human, i.e. human and macaque (B) cortical wall at peak stages of neurogenesis, and neurons accumulating in the cortical plate.

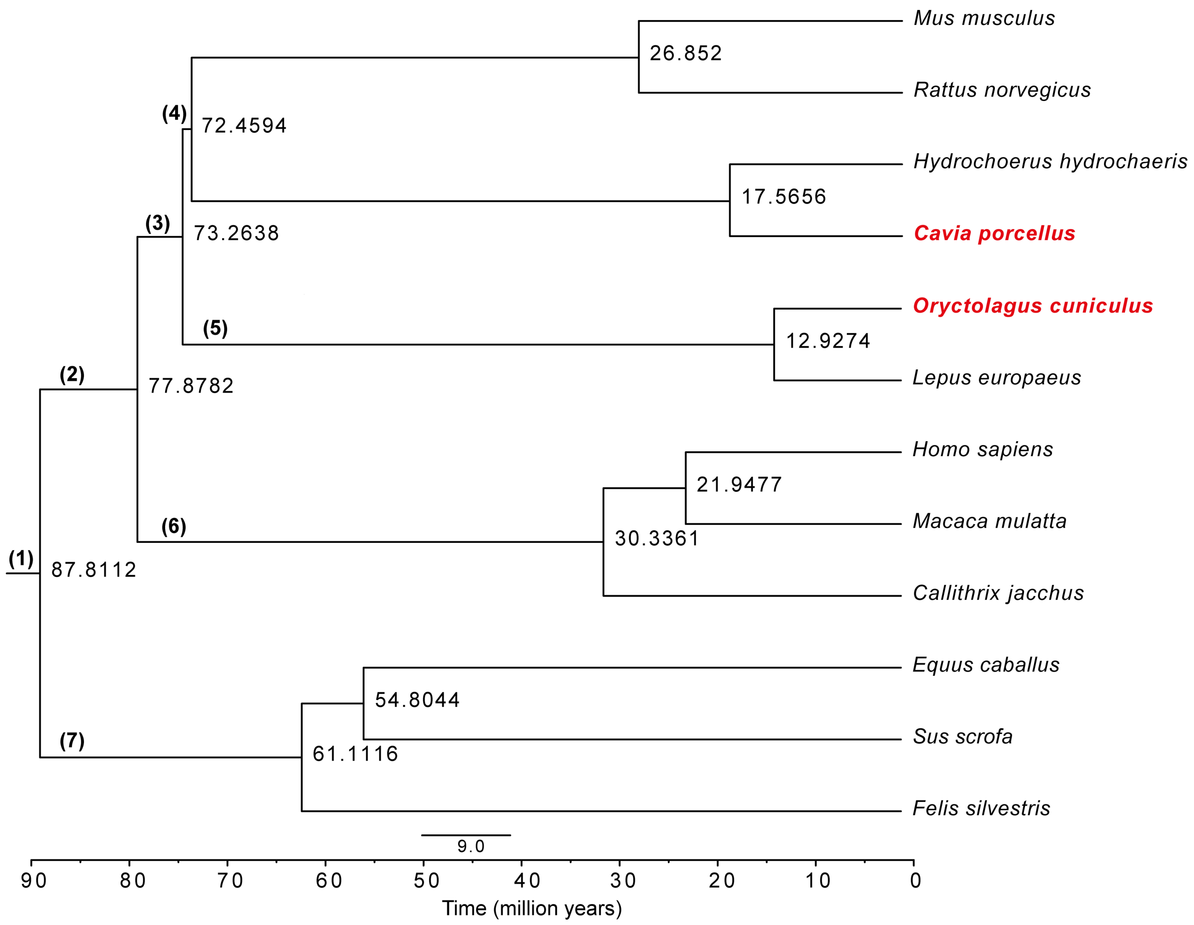

SUPPLEMENTARY FIGURE 2 | Phylogenetic relationship between the guinea pig and rabbit and related taxons. Cladogram depicting the evolutionary relationship between the following mammal species: *Rattus norvegicus, Mus musculus, Hydrochoerus hydrochaeris, Cavia porcellus, Oryctolagus cuniculus, Lepus europeus, Homo sapiens, Macaca mulatta, Callithrix jacchus, Sus scrofa, Equus caballus, Felis silvestris*. Species used in this study are highlighted in red. Numbers in brackets refer to the following taxons: 1, Boreoeutheria; 2, Euarchontoglires; 3, Glires; 4, Rodentia; 5, Lagomorpha; 6; Euarchonta; 7, Laurasiatheria. The tree was generated using the MammalTree service from vertlife.org and Figtree1.4.4. For details, see Materials and Methods. Scale in million years.

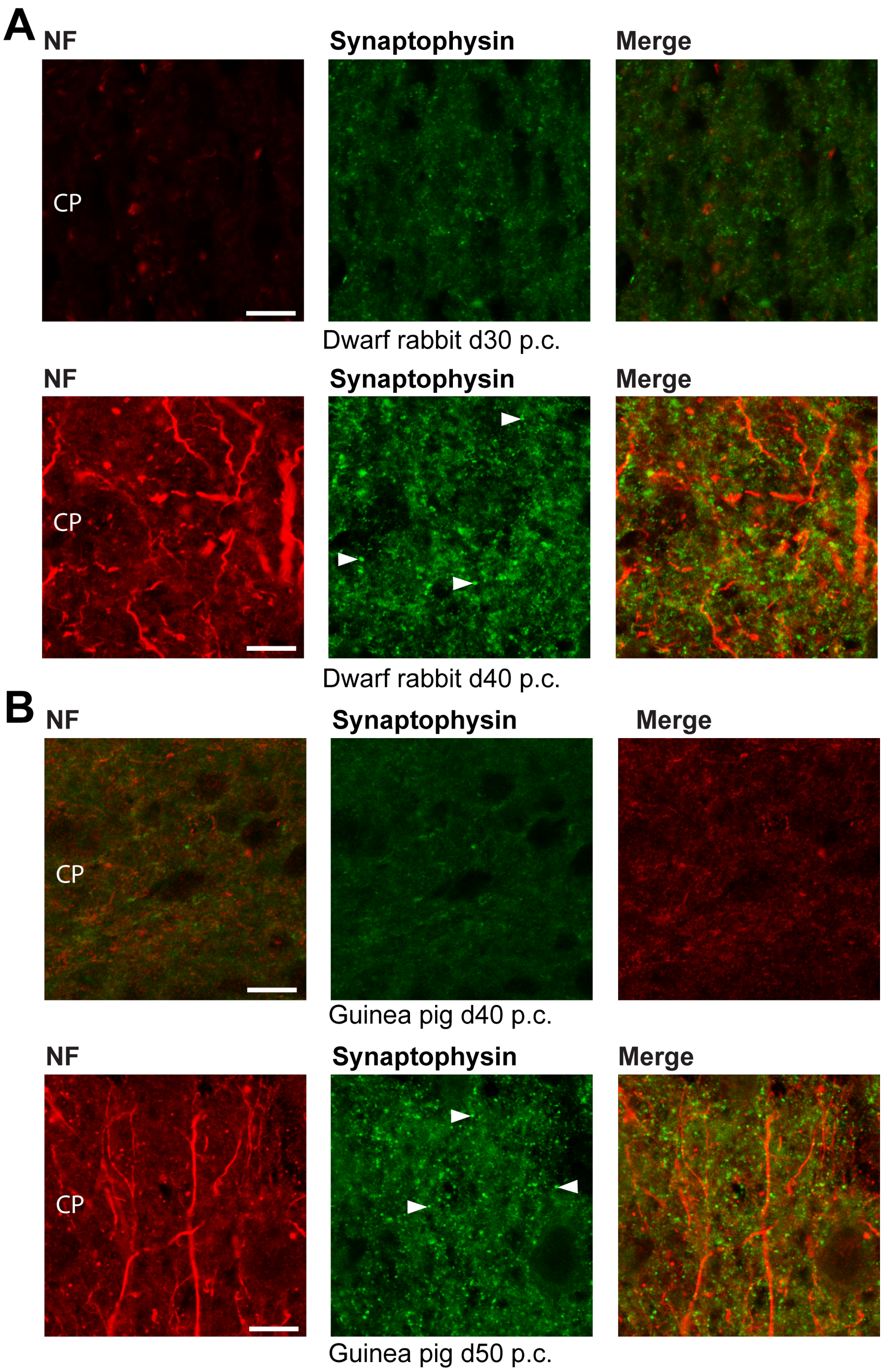

SUPPLEMENTARY FIGURE 3 | Neurofilament H and synaptophysin expression in the developing dwarf rabbit and guinea pig cortical plate (CP). Double-Immunofluorescence for neurofilament (NF, red) and synaptophysin (green) on 30 µm-cryosections of d30 and 40 p.c. dwarf rabbit and d40 and 50 p.c. guinea pig CP. Merge images show combined immunofluorescence of NF and synaptophysin. Solid arrowhead, presynaptic vesicles. Scale bars, 10 µm.
